# Household preparedness for emergencies during COVID-19 pandemic among the general population of Nepal

**DOI:** 10.1371/journal.pgph.0003475

**Published:** 2024-09-12

**Authors:** Salina Shrestha, Rabin Malla, Sadhana Shrestha, Pallavi Singh, Jeevan B. Sherchand

**Affiliations:** 1 Center of Research for Environment, Energy and Water, Kathmandu Nepal; 2 Interdisciplinary Center for River Basin Environment, University of Yamanashi, Yamanashi, Japan; 3 Nepal Red Cross Society, Head Office, Kathmandu, Nepal; 4 Institute of Medicine, Research Directorate, Tribhuvan University, Kathmandu, Nepal; Nanyang Technological University, SINGAPORE

## Abstract

The COVID-19 pandemic has negatively impacted the global economy affecting numerous people’s livelihoods. Despite preventive behaviors and advancements of vaccination, the risk of infection still exists due to the emergence of new variants of concern and the changing behavior of the SARS CoV-2 virus. Therefore, preparedness measures are crucial for any emergency. In such situations, it is important to understand preparedness behavior at the household level, as it aids in reducing the risk of transmission and the severity of the disease before accessing any external support. Our study aimed to evaluate household preparedness level for emergencies during the COVID-19 pandemic and its relationship with socio-demographic characteristics among the general population of Nepal. Data was collected through a questionnaire survey. Descriptive statistics, a Chi-square test, and logistic regression model were used for analysis. The study demonstrated that 59.2% had a good preparedness level. Good preparedness was observed among the respondents living in urban areas, those who were married, had white-collar occupations, high-education with graduate and above and high-income levels with monthly income >NPR 20,000, and were young-aged. The study findings underscore the need to develop tailored programs on preparedness prioritizing vulnerable population. It further highlights the importance of proper and consistent information flow, resources distribution, capacitating human resources and better health surveillance.

## Introduction

The sudden onset of emergencies has resulted in a vivid increase in damage to life and property over the last few decades, leading to severe health impacts [1]. Coronavirus disease 2019 (COVID-19) has become one of the major public health issues globally and was declared a pandemic on 11 March 2020. It transmitted rapidly to almost all the countries of the world compared to the previous coronavirus epidemics such as SARS (Severe Acute Respiratory Syndrome) and MERS (Middle East Respiratory Syndrome) over the past three years [2]. The pandemic has affected the global economy and affected the livelihoods of many people [3]. As of 24 December 2023, the total number of confirmed COVID-19 cases worldwide was around 773,119,173 with a death toll of 6,990,067 [4]. In Nepal, there were a total of 1,003,450 reported confirmed COVID-19 cases and 12,031 deaths [5].

Recently, the World Health Organization (WHO) lifted the declaration of the public health emergency of international concern due to diminishing waves of COVID-19. However, experts have made it clear that the threat still exists, and the pandemic is not over. The disease remains and people must learn to live with it, as in the case of influenza, cholera etc. where the virus cannot be eradicated [6, 7]. Hundreds of thousands of cases, along with numerous deaths, are still occurring [8]. A considerable number of people are suffering from long COVID-19 [9], and higher risks exist among individuals with chronic diseases [10]. At this moment, the major concern is the changing nature of the virus possessing uncertainties and more variants are still expected to evolve with no inevitability about changes in severity. This chaotic situation underscores the necessity of always being in a state of readiness. During this phase, WHO advises countries not to let down its guard and to maintain systems built during the challenging times of the pandemic. The Global Strategic Preparedness, Readiness and Response Plan for the period 2023–2025 [11], updated by WHO, further suggests incorporating and continuing to monitor COVID-19 cases through community-based surveillance in national programs. It strongly emphasizes data collection and reporting in various aspects of this matter to reduce infection.

The crucial matters to be considered for inquest to control the disease are, firstly, measures followed by non-infected individuals for prevention; secondly, preparedness measures required prior to getting infected. Despite awareness, adherence of preventive practices, and the advancements in vaccinations, the risk of emergencies, such as the possibility of any family members getting infected by COVID-19, still exists. In such situations, compliance with preparedness measures is crucial and plays a special role in lessening the deleterious impact of resurging COVID-19. For example, huge loss of life and property can be prevented through strong preparation before the onset of emergencies in the family [12]. Therefore, studying preparedness is crucial.

Preparedness is usually considered as the ‘knowledge and capacities to effectively anticipate, respond and recover from the impact of hazard events’ [13]. It should be addressed at different levels, including household, community, and government levels. Household preparedness is especially important in such situations, as it involves the preparation for self-rescue measures that need to be conducted immediately and appropriately before accessing other external support [14]. These measures comprise hygiene, proper isolation, effective communication, and storage of items including medicine, food, water, hygiene material, etc. during emergencies [15–17]. The proper home quarantine of family members taking care of the infected person, should also be preferred [18, 19]. A lack of preparedness prior to the onset of any emergency usually makes people anxious and distressed due to fear of the unknown, that negatively influences their ability to make decisions and causes difficulties in taking the right actions during emergencies [20–23]. Therefore, awareness of the household-level preparation is crucial to ease the panic and lessen the severity of the crisis. Understanding of preparedness among the people is indispensable for this reason.

Household preparedness is crucial and needs to be prioritized, especially in developing countries like Nepal, to reduce the burden on health institutions with inadequate resources [24–26]. Additionally, it helps to enhance the adequate management of crisis within households. Numerous awareness programs for prevention from COVID-19 are conducted by many organizations [27, 28]. However, the dissemination of information on several aspects of household preparedness for emergencies is comparatively less frequent. Moreover, the inequality in access to information, and required resources regarding the adversity of emergencies is ubiquitous in relation to variation in socio-demographic status such as geographical location, marital status, gender, education, income, occupation [29–33]. This further diminishes the perceived severity of emergencies among the people with limited access and exhibits the possibility of variation in preparedness behavior with respect to socio-demographic characteristics. Nevertheless, it is imperative to increase the perceived severity to motivate people to act at any time with careful measures that determine preparedness behavior [34].

Previous studies on household preparedness for emergencies in different countries have revealed diverse information that aids in comprehending the status of preparedness behavior, its influencing factors and provides the guidelines for policy recommendations [14, 19, 35–40]. Nonetheless, the lack of detailed studies on household preparedness for emergencies in the context of Nepal accentuates the importance of this study. We assumed differences in compliance with household preparedness measures among the general population with reference to socio-demographic status. Therefore, this paper intends to assess the situation of household preparedness for emergencies during COVID-19 pandemic at the household level and its relationship with socio-demographic characteristics. The study results will provide empirical evidence that can help to revise the existing plan and policies, apportion the required resources as well as its efficient use during emergencies to improve preparedness. Additionally, understanding the variation in preparedness according to social and demographic factors in society will give a clear picture of the vulnerable population.

## Methods

### Study area and sampling

**Fig 1:**
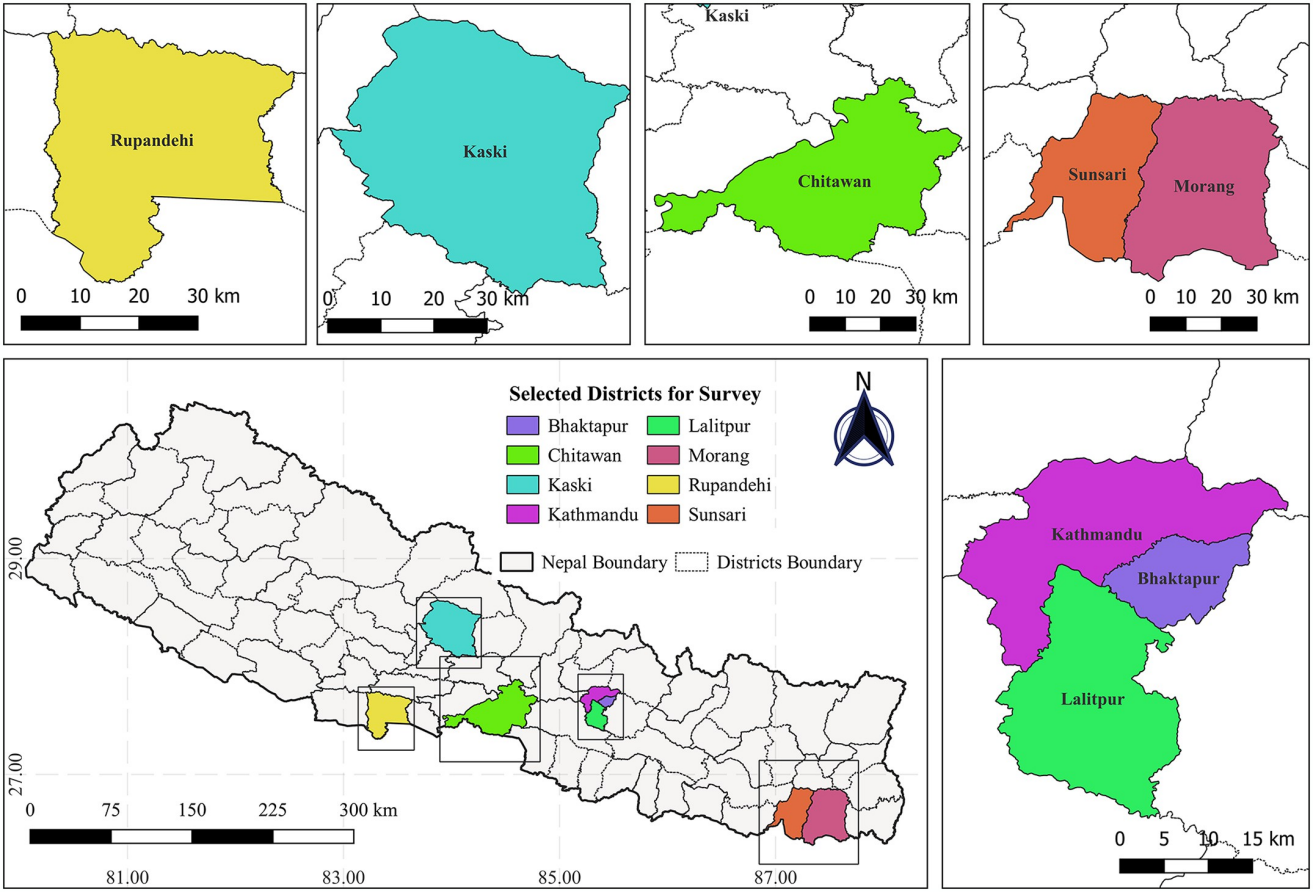
Location of the selected districts (Bhaktapur, Chitwan, Kaski, Kathmandu, Lalitpur, Morang, Rupandehi, and Sunsari). [The darkest grey thick lines covering the grey area represents the country boundary of Nepal and the soft grey lines inside the country boundary represent the administrative boundaries of Nepal separating different districts. The location of selected districts for the study is presented by rectangles. Country boundary and administrative boundaries shape files of Nepal, retrieved from Administrative Boundaries Reference (view layer) of FAO (https://data-in-emergencies.fao.org/datasets/hqfao::administrative-boundaries-reference-view-layer/explore?layer=0), under an open license (CC BY 4.0): https://data-in-emergencies.fao.org/datasets/hqfao::administrative-boundaries-reference-view-layer/about?layer=0. Figure created with QGIS software version 3.28, an open source Geographic Information System (GIS) licensed under the GNU General Public License (https://bit.ly/2BSPB2F)]. *Source: Shrestha et al. 2023* [41].

A cross-sectional study was conducted in eight of seventy-seven districts of Nepal which were the most affected districts during the COVID-19 pandemic—Kathmandu, Bhaktapur, Lalitpur, Morang, Sunsari, Rupandehi, Chitwan, and Kaski (Fig 1). Eight districts were chosen to include 10% of the total districts of the countries. The study considered the general population above the age of 18 years who could give consent to participate in the survey. During the period of the pandemic, due to the difficulties of conducting probability sampling, a convenient non-probability sampling method was considered for data collection. The total sample size was 702.

For each selected district, the sample size was determined according to the population proportion of the total population of eight districts. The populations of the selected districts are as follows; Kathmandu—1,744,240, Bhaktapur—304,651, Lalitpur—468,132, Morang—965,370, Sunsari- 763,487, Rupandehi—880,196, Chitwan—579,984, and Kaski—492,098 [42].

The sample size for each district was calculated as follows:

Proportionofpopulationineachdistrict(A)=(Populationofadistrict)/(Totalpopulationofalleightdistricts)×100%.

$$\text{Sample}\;\text{size}\;\text{of}\;\text{each}\;\text{district}\;({\text{n}}_{\text{d}})\;=\;(\text{A})/100\;\times \;\text{Total}\;\text{sample}\;\text{size}\;(702).$$

Hence, the numbers of participants selected in each district were as follows: Kathmandu—197, Bhaktapur—35, Lalitpur– 54, Morang—107, Sunsari—88, Rupandehi—99, Chitwan—66, and Kaski—56.

The study was conducted from 21 March 2021 to 12 April 2021. The same survey population was also considered to analyze the knowledge, attitude and practices on COVID-19 [41].

### Ethical concern

The Ethical Review Board, Nepal Health Research Council approved the study, and the ethical approval number is Ref No. 2240. The interviewers explained the study details, including its objectives and significance. They assured respondents that their identities would remain confidential and emphasized their freedom to stop participating in the survey at any time, as participation was voluntary. Informed consent was obtained from all the respondents before the survey began. Verbal informed consent was obtained from respondents who contributed via telephone survey and included as recording, and written informed consent was obtained from those who contributed via face-to-face survey.

### Measurements

#### Preparedness

Eight binary (yes/no) questions were used to measure the household preparedness level of the general population for emergencies during the COVID-19 pandemic. The questions included: (1) confidence in coping with any situation during the pandemic, (2) availability of sufficient space for isolation, (3) availability of a well-ventilated room, (4) good knowledge of daily safety measures, (5) management of sufficient food and money to cope with any situation, (6) preparation of a list of contact numbers for the police, ambulance, and hospital in case of any emergency, (7) trust among friends, neighbors, and relatives for their cooperation if any family member gets infected, and (8) taking care of the mental health of family members. The total score of preparedness ranged from 0–8, with a cut-off value of 7 (median). The score values of 0–6 were categorized as poor preparedness and 7 to 8 were categorized as good preparedness.

#### Socio-demographic characteristics

The information on socio-demographic characteristics of households was collected using a structured questionnaire that included the following variables: geographical location (rural municipality, urban municipality), gender (male, female), age (<20 years, 20–30 years, 31–40 years, 41–50 years, >50 years), marital status (married, unmarried), education (no education, literate, basic education, secondary education, undergraduate, graduate and above), income (<NPR 5000, NPR 5000–10,000, NPR 10,000–15,000, NPR 15,000–20,000, NPR >20,000) (USD 1 = NPR 133.12), occupation of the respondent (white-collar occupation—service, business, house rent; blue-collar occupation—agriculture, labor; and others—self-employed, remittance and others).

The questionnaire on preparedness was developed after reviewing prior studies from Hong Kong [37], and various other relevant sources [15–17, 43, 44]. Experts translated the questionnaire, which was developed in English language initially, translated into Nepali language and then back translated it to English. A pre-test of the questionnaire was conducted among 30 respondents before the administration of the final version. The questionnaire was finalized after including the suggestions received from the pre-test, ensuring clear vocabulary and simple sentence structure.

### Statistical analysis

Descriptive statistics were utilized to calculate the frequency, proportion and median. The Chi-square test was employed to assess the differences in household preparedness levels in relation to socio-demographic status. Binary logistic regression was used to predict the relationship between household preparedness level (dependent variable) and socio-demographic variables (independent variables). Cronbach’s Alpha (α) was used to measure the reliability of the preparedness questionnaire. The significance level for all the statistical analysis was set at 5%. Statistical analyses were conducted using Statistical Package for the Social Sciences V.20 (SPSS Inc, Chicago, Illinois, USA).

## Results

### Preparedness

The study revealed that, 95.1% (662) of respondents had taken care of the mental health of their family members, 91.7% (640) were confident about their coping ability with any situation during the pandemic, 88.7% (618) had well-ventilated room, 85.7% (598) were aware on daily safety measures to be considered and 83.8% (585) were confident in receiving help from friends, neighbors and relatives. On the other hand, only 72.5% (506) had managed sufficient food and money for emergency situations, 67.9% (474) had sufficient space for isolation and 63.2% (441) had prepared a list of contact numbers for emergencies (Table 1). Among the total respondents, 59.2% (411) had good preparedness and 40.8% (283) had poor preparedness.

**Table 1:** Preparedness for emergencies during the COVID-19 pandemic among the respondents.

S.N.	Items	Yes (%)
PRD1	I am confident that I can cope with any situation during the pandemic	640 (91.7%)
PRD2	If any of the family members are infected, we have sufficient space for isolation.	474 (67.9%)
PRD 3	We have a well-ventilated room	618 (88.7%)
PRD 4	I have good knowledge of daily safety measures to be considered	598 (85.7%)
PRD 5	I have managed sufficient food and money to cope with any situation during the pandemic	506 (72.5%)
PRD 6	I have prepared a list of contact numbers for the police, ambulance, and hospital in case of any emergency.	441 (63.2%)
PRD 7	I believe that my friends, neighbors, and relatives will cooperate if any family member gets infected	585 (83.8%)
PRD 8	I have taken care of the mental health of my family member	662 (95.1%)

### Relationship of preparedness and socio-demographic characteristics

The α value of the questionnaire for measuring preparedness on COVID-19 was 0.76. Our study illustrated that proportion of respondents with good preparedness was significantly higher among respondents residing in urban areas, at 61.3% (383), compared to those residing in rural areas, at 38.1% (24) (p<0.001). Unmarried respondents also exhibited significantly higher levels of good preparedness, at 71.8% (155), compared to married respondents, at 53.6% (255) (p<0.001). Similarly, the higher proportion of respondents with white-collar occupations demonstrated good preparedness, at 69.6% (289), compared to those with blue-collar occupations, at 30% (36), and other occupations, at 53.2% (66) (p<0.001). Regarding the relationship with education, an increasing educational level showed an increase in preparedness. Respondents with high-education level of graduate and above had significantly good preparedness level, at 87% (60), compared to other respondents (p<0.001). Additionally, the high-income group, with a monthly income of >NPR 20,000, demonstrated significantly good preparedness, at 76.4% (343), compared to those with other low-income groups (p<0.001). In the same way, the proportion of respondents with good preparedness was significantly higher among the younger age group of <20 years, at 81.8% (27), compared to other age groups (p = 0.01) (Table 2).

**Table 2:** Socio-demographic characteristics and the proportion of the population with preparedness for emergencies during the COVID-19 pandemic.

Variables	Number of respondents	Good preparedness (%)	Chi-square test (p-value)
**Municipality**			
Rural	63	24 (38.1%)	<0.001
Urban	625	383 (61.3%)	
**Gender**			
Male	330	205 (62.1%)	0.13
Female	364	206 (56.6%)	
**Marital status**			
Unmarried	216	155 (71.8%)	<0.001
Married	476	255 (53.6%)	
**Main occupation**			
Blue-collar	120	36 (30%)	<0.001
White-collar	415	289 (69.6%)	
Others^a^	124	66 (53.2%)	
**Age (years)**			
<20	33	27 (81.8%)	0.01
20–30	256	163 (63.7%)	
31–40	158	86 (54.4%)	
41–50	136	77 (56.5%)	
>50	109	57 (52.3%)	
**Education of respondent**			
No Education	54	10 (18.5%)	<0.001
Literate	23	10 (43.5%)	
Basic Education	89	27 (30.3%)	
Secondary Education	226	126 (55.8%)	
Undergraduate	230	176 (76.5%)	
Graduate and above	69	60 (87%)	
**Income-Nepalese rupees (NPR)**			
<5000	29	5 (17.2%)	<0.001
5000–10,000	48	7 (14.6%)	
10,000–15,000	61	14 (23%)	
15,000–20,000	104	42 (40.4%)	
>20,000	449	343 (76.4%)	

^**a**^ Others—self-employed, remittance and others

The logistic regression analysis showed that the respondents with education level of graduate and above (OR: 10.06, 95% CI 3.08 to 32.91), undergraduate (OR: 5.97, 95% CI 2.22 to 16.05) and secondary education (OR: 4.61, 95% CI 1.80 to 11.77) had higher odds of having good preparedness compared to non-educated respondents. Additionally, respondents with high-income (>NRs 20,000) were 10.33 times more likely to have good preparedness (95% CI 3.05 to 34.91) than those with low-income. Regarding the age groups, respondents aged 20–30 years (OR: 0.20, 95% CI 0.06 to 0.71), 31–40 years (OR: 0.20, 95% CI 0.05 to 0.78) and 41–50 years (OR: 0.21, 95% CI 0.05 to 0.84) had lower odds of having good preparedness compared to young-aged respondents (<20 years) (Table 3).

**Table 3:** Odds ratios (OR) on preparedness for emergencies during the COVID-19 pandemic among the general population, according to socio-demographic characteristics.

Variables	Univariate	Multivariate
**Municipality**		
Rural	1	1
Urban	2.52 (1.50 to 4.38)**	1.73 (0.91 to 3.30)
**Gender**		
Male	1	1
Female	0.79 (0.58 to 1.07)	0.82 (0.55 to 1.23)
**Marital status**		
Unmarried	1	1
Married	0.54 (0.31 to 0.64)***	0.90 (0.50 to 1.64)
**Main occupation**		
Blue-collar	1	1
White-collar	5.35 (3.43 to 8.33)***	1.64 (0.97 to 2.95)
Others	2.65 (1.56 to 4.49)***	1.24 (0.65 to 2.36)
**Age (years)**		
<20	1	1
20–30	0.38 (0.15 to 0.97)*	0.20 (0.06 to 0.71)*
31–40	0.26 (0.10 to 0.67)**	0.20 (0.05 to 0.78)*
41–50	0.29 (0.11 to 0.74)*	0.21 (0.05 to 0.84)*
>50	0.24 (0.09 to 0.63)**	0.32 (0.07 to 1.34)
**Education of respondent**		
No Education	1	1
Literate	3.38 (1.15 to 9.89)*	2.38 (0.66 to 8.55)
Basic Education	1.91 (0.84 to 4.35)	1.78 (0.65 to 4.82)
Secondary Education	5.54 (2.65 to 11.56)***	4.61 (1.80 to 11.77)**
Undergraduate	14.34 (6.76 to 30.39)***	5.97 (2.22 to 16.05)***
Graduate and above	29.33 (10.99 to 78.23)***	10.06 (3.08 to 32.91)***
**Income-Nepalese Rupees (NPR)**		
<5000	1	1
5000–10,000	0.82 (0.23 to 2.87)	1.36 (0.32 to 5.76)
10,000–15,000	1.43 (0.46 to 4.44)	1.69 (0.44 to 6.44)
15,000–20,000	3.25 (1.14 to 9.20)*	3.11 (0.88 to 10.94)
>20,000	15.53 (5.78 to 41.71)***	10.33 (3.05 to 34.91)***

* p < 0.05,

** p < 0.01,

*** p < 0.001

## Discussion

Our study assessed the preparedness of the general population for emergencies during the COVID-19 pandemic and its association with socio-demographic characteristics.

The findings revealed that many of the respondents were still not considerate about good household preparedness measures. Studies conducted in three provinces [35] and four regions [14] of China also showed a lower proportion of study population, with only 28% and 9.9% adhering to household preparedness measures, respectively. Similarly, 59.2% of total respondents in Hong Kong adhered to good household preparedness during an emergency, indicating that many are still at risk [37].

Our study uncovered the fact that a substantial proportion of respondents had well-ventilated rooms in their houses. Previous studies conducted in Spain [45], China [46], United Kingdom and Italy [47] also revealed access to improved ventilation in households among people after the pandemic started. In the indoor household setting, the aerosols containing SARS COV-2 virus generated by sneezing, coughing and talking by infected persons can increase the risk of transmission. Proper ventilation allows the fresh air to move in and quickly remove the virus from the room, reducing the risk [48]. However, the lack of sufficient space for isolation among the respondents in our study can further increase the threat. The aforementioned study also reported the dearth of space in many of the households for isolation [49]. Small households usually face more difficulties in managing separate spaces for isolation [50]. Despite the large number of infected people who stayed in home isolation [49], adherence to safety measures cannot be assured and is not monitored regularly [51]. The virus shedding, frequently touched surfaces, and deposition of respiratory droplets in the room of an infected person pose a risk of fomite transmission [52]. Therefore, it is necessary to avoid the crowded environment in the room and allocate a room for isolation to reduce the infection risk. The establishment of isolation centers in every locality can help address such issues.

The study found that a significant number of respondents were knowledgeable about the daily safety measures required for the prevention of COVID-19 transmission. This knowledge is especially important in the closed environment of the household setting, where frequent contact occurs. The household setting possesses a higher risk of secondary transmission attributable to the survival duration of the virus in aerosols and fomites compared to non-household settings [53–55]. The importance of good knowledge and practices for safety measures and prevention was also highlighted in the prior studies [14, 56, 57]. Key safety measures, such as physical distancing, proper use and disposal of personal protective equipment (PPE), hand hygiene, cleanliness of contaminated surfaces and other necessary stuffs, use of dedicated utensils for the infected person etc. inside the home are imperative for breaking the chain of transmission of SARS CoV-2 and play a key role in reducing the spread of the disease [43, 44, 58]. Compliance with safety measures requires adequate facilities of Water, Sanitation and Hygiene (WASH) [59–61] and easy access to PPE at affordable prices [62].

A considerable number of respondents in our research had managed food and money for emergencies compared to the aforementioned studies. For instance, only 6.7% of the study population in four regions of China (Beijing, Guangdong, Heilongjiang and Sichuan) [4] and 31.1% in three provinces of China (Heilongjiang, Guangdong and Sichuan) [35] had managed food and money for emergencies. Similarly, in Hong Kong 57.3% of the population reported having sufficient food items [63]. Food is a life saver and the most important item required for preparedness during emergencies. Almost everyone should store preferably non-perishable food for emergencies. Moreover, saving money for emergencies help protect against financial crisis arising from several unexpected circumstances such as extra expenses for frequent hospital visits and treatments, loss of income, and job etc. Savings can help manage crisis, increase resilience, and provide support to easily bounce back from such difficult situations.

Our study demonstrated that a significant proportion of respondents had not prepared a list of contact numbers of the police, ambulance, and hospital in case of emergencies. Although several hotline numbers have been made available by concerned organizations to inquire about COVID-19 during emergencies [44], a considerable portion of study population were unaware of using these numbers. Conversely, a similar study conducted in Saudi Arabia revealed the contrasting results, showing increased awareness and the number of emergency calls among the people during the pandemic [64]. The health condition of COVID-19 patients can further worsen, increasing the risk of death, if they are kept at home during the sudden onset of severe symptoms. The situation is further aggravated if the person possesses other chronic illnesses [10]. In such situations, immediate calls for ambulance, hospital or police are important for timely treatment and reducing risk. Improving familiarity with existing hotline numbers and understanding the significance of using them is crucial.

On the other hand, among the total respondents, the majority were more confident about receiving support from friends, neighbors, and relatives, indicating that a substantial number had good social capital in terms of a social network. A study conducted in China also revealed the similar result, with a significant proportion of study population having high social support during COVID-19 pandemic [65]. Social capital is underscored as the strongest factor of preparedness, which helps to control the spread of disease and maintain the mental and physical health of people [66, 67]. Social capital, in relation to the social network, such as the presence of trustworthy friends and relatives, also increases the confidence in dealing with difficult situations during emergencies, especially regarding financial and physical help [39, 68, 69]. Therefore, preparing a list of contact numbers of appropriate family and friends should be prioritized. The use of digital media is one of the most effective ways to improve social networks even in times of crisis.

The study showed that a significant proportion of respondents were confident about their ability to cope with any situation during the pandemic, and majority of them were observed to be conscious about taking care of the mental health of their family members. The key point to note is that mental health problems are prevalent globally [70], including in Nepal during the pandemic [71, 72]. The earlier studies also highlighted that a substantial number of people staying with family members who receive care and support are comparatively less susceptible to mental health problems such as stress and depressive disorders etc. further supporting our study findings [72, 73].

Our study also disclosed that the preparedness of the general population was associated with socio-demographic characteristics. The study population from rural areas was observed to be less prepared compared to those from urban areas, which resembles the study conducted in China [14]. The lack of resources and weak communication channels in rural areas, which hamper the adequate and timely flow of information, might be the possible reason for poor preparedness [30, 74]. This fact was justified by a study conducted in Thailand, which showed 1.35 times increase in the preparation for emergencies with the addition of each source of information [75]. Similarly, a lower proportion of married respondents had good preparedness compared to unmarried respondents, though this became statistically insignificant under a 5% significance level in the logistic regression when conditioned on other variables. Our study further illustrated that respondents with higher education were more prepared for emergencies. Study conducted in Hong Kong [37], Serbia [40], and Thailand [75] also revealed an association of good preparedness with high educational background. Generally, educated people acquire more knowledge and are more frequently updated. Their better intellectual ability, processing of acquired knowledge, and propensity for learning equip them to understand the severity of risks and make them aware to comply with preparedness measures. Accordingly, they can properly allocate resources and plan for future emergencies [75]. The preparedness for emergencies was higher among respondents with white-collar occupations, which bears a resemblance to the study conducted in Hong Kong [37]. Regarding income level, a significant proportion of respondents with high-income had good preparedness and were more likely to practice the necessary measures. Our finding aligns with the study conducted in Hong Kong [37]. The reason might be that people with high incomes and white-collar occupations feel more secure, have sufficient resources, and have access to necessary items required for preparedness. Concerning age, youngest respondents showed good preparedness compared to old respondents. Additionally, the decline in statistical significance for Age > 50 noted from univariate to multivariate logistic regression could be because of the lower education levels amongst the older generation i.e. a potential confounding effect. A previous study showed that most with older age are illiterate or less educated due to lack of opportunities and family obligations in earlier days [76]. The higher use of internet and social media among the young-aged people might be one of the possible reasons for their good preparedness. Online platforms and social media are usually considered powerful mediums for rapid flow of information from different parts of the world, aiding in awareness and behavioral change [77]. Nonetheless, contrasting results were observed in a study conducted in Texas, USA, which showed good preparedness among older aged people [78]. The mixed results of different studies regarding the age factor underscore the necessity of detailed studies on the underlying reasons for in-depth understanding.

Enhancing the adherence of preparedness measures for health emergencies among the community people is paramount, especially during a health crisis. Prompt reporting of sickness if any family member becomes infected is of utmost importance to reduce both the severity of the disease and its transmission. However, fear of stigmatization often inhibits the disclosure of sickness to the concerned authorities [79].

Several policy implications are listed below in Table 4.

**Table 4:** Policy implications for the population lacking household preparedness.

Issues	Policy implications
Lack of confidence to cope with any situation during the pandemic	Empowering people to handle any difficult situation during a crisis through awareness programs aimed at improving problem-solving skills.
Lack of sufficient space for isolation.	Implementing a robust health surveillance system, including contact tracing and locating infected individuals, monitoring the status of isolation at the household level to handle cases effectively. Establishing isolation centers in various localities equipped with well-ventilated rooms and all required facilities as needed. Insisting that people stay in centers if insufficient space at home to reduce the risk of infection Prioritizing regular reporting of disease control activities by the government to cultivate trust among the community and enhance individual self-discipline in adhering to necessary measures and reporting sickness at the earliest, to the concerned authorities.
Lack of well-ventilated room	
Lack of good knowledge regarding daily safety measures	Extensively disseminating resources and guidelines prepared by the government, such as the ‘Pocket book for the people in home isolation’ (44), ‘COVID-19 case isolation management guidelines’ (15), ‘Health standards for isolation among COVID-19 infected patient 2020’ (17) and circulating important information comprised within through different media. This includes the importance of quarantine, isolation and adherence to preparedness measures at household level, such as storing essential items, hygiene activities, helpline numbers etc. and regular monitoring by concerned authorities which can foster household preparedness. Distributing essential supplies to affected families, required for daily purposes including food, money, hygiene materials etc., motivates people to follow household preparedness with the home isolation and quarantine properly.
Not managed sufficient food and money to cope with any situation during the pandemic	
A list of contact numbers for the police, ambulance, and hospitals is not well-prepared for any emergency.	Disseminating information regarding hotline numbers including police, ambulance and hospitals on a wider scale
Lack of confidence that social relations, such as friends, neighbors, and relatives will provide help and cooperate if any family member gets infected.	Raising awareness among people about the importance of strengthening their social capital and networks, and encouraging mutual support within the community during a crisis
Low prioritization to mental health	Planning and implementing awareness programs to equip people with the skill to take care of their own and their family members’ mental health during a crisis. Assigning an adequate number of experts to provide prompt and accessible mental health services to the entire population
Variation in household preparedness behavior with respect to socio-demographic characteristics	Planning specific programs that consider diverse socio-demographic conditions, to improve preparedness among the general population. Comprehensive risk communication through different media, targeting individuals with various socio-demographic characteristics, should be specially prioritized. To reach the less educated and older people, information should be disseminated through TV and radio in understandable language. Community health workers should also be mobilized at the local level to aware them. Special packages on adult education and digital literacy are essential to enable them to access the timely information.
Proper co-ordination and allocation of roles, responsibilities and resources among government bodies and other responsible organizations, along with their accountability for implementing preparedness measures effectively is crucial. Capacity building of human resources involved in improving household preparedness is indispensable for the successful implementation of the intervention programs. Establishing robust monitoring and evaluation mechanisms to assess the effectiveness of intervention programs and make necessary improvements.	

There are several limitations in the study. The study is cross-sectional, that gives only a snapshot of the characteristics of the population. In addition, telephone surveys were also conducted in some areas that were difficult to reach during pandemic for face-to-face survey. The association of social norms, ethnicity, religious and cultural diversity with preparedness behavior among the general population were not included in the study. A future study considering these factors during health crisis is critical for comprehensive understanding. Moreover, detailed investigations on the hurdles in following preparedness measures and identifying motivating factors for positive behavior change is crucial.

## Conclusion

The study put forward a comprehensive analysis of household preparedness during the COVID-19 pandemic among the general population and its influencing factors. A significant proportion of respondents demonstrated poor preparedness at the household level for emergencies. Many lacked sufficient space for isolation in their homes, did not prepare a list of contact numbers of the police, ambulance and hospital and did not manage sufficient food and money to cope with the crisis. Good household preparedness levels were mostly observed among the respondents living in urban areas, married individuals, those with white-collar occupations, young, aged groups, high-education level with graduate and above and high-income level with monthly income >NPR 20,000.

To address this issue, a tailored program on household preparedness for the pandemic, emphasizing vulnerable groups, is crucial. Consistent and timely flow of information through various communication channels regarding the possible severity of the disease, importance of household preparedness measures along with better health surveillance aids in dealing with the health crisis. Additionally, building trust in the government is essential to ameliorate the people’s self-discipline in adhering to adequate measures.

## Supporting information

S1 Questionnaire (DOCX)

S1 Data (XLSX)

S1 Table Characteristics of the study population. (DOCX)
