# Supplementary material for: Household preparedness for emergencies during COVID-19 pandemic among the general population of Nepal
Source: PLOS Glob Public Health. 2024 Sep 12;4(9):e0003475. doi: 10.1371/journal.pgph.0003475 (PMC11392347; doi:10.1371/journal.pgph.0003475)
Supplement: S1 Questionnaire — (DOCX) [file pgph.0003475.s001.docx]

**Questionnaire**

Sample ID: ………………………………………………

Name: ………………………………………………………..

Address:…………………….………Province……………………..……District ………………………………Municipality

Contact no…………………………………………….

**A. Personal information**

**Q.N.1** What is your gender?

i) Male ii) Female

**Q.N.2** What is your marital status?

i) Married ii) Unmarried

**Q.N.3** Please mention about the major occupation.

i) Agriculture ii) Service iii) Business iv) Labor v) House rent vi) Self-employed vii) Remittance viii) Others ………………………

**Q.N.4** What is your age?

i) <20 years ii) 20-30 years iii) 31-40 years iv) 41-50 years v) >50 years

**Q.N.5** What is your highest education?

I) No Education ii) Literate iii) Basic Education iv) Secondary Education v) Undergraduate vi) Graduate & above

**Q.N.6** Please mention about the monthly income.

i) <NPR 5000 ii) NPR 5000-10,000 iii) NPR 10,000-15,000 iv) NPR 15,000-20,000 v) NPR >20,000

**B. *Preparedness***

**Q.N.7 Do you adhere to the following preparedness measures? Please choose the appropriate answers. (√)**

| **Items** | **Yes** | **No** |
| --- | --- | --- |
| I am confident that I can cope with any situation during the pandemic |  |  |
| If any of the family members are infected, we have sufficient space for isolation. |  |  |
| We have a well-ventilated room |  |  |
| I have good knowledge of daily safety measures to be considered |  |  |
| I have managed sufficient food and money to cope with any situation during the pandemic |  |  |
| I have prepared a list of contact numbers for the police, ambulance, and hospital in case of any emergency. |  |  |
| I believe that my friends, neighbors, and relatives will cooperate if any family member gets infected |  |  |
| I have taken care of the mental health of my family member |  |  |
