# Supplementary material for: Household preparedness for emergencies during COVID-19 pandemic among the general population of Nepal
Source: PLOS Glob Public Health. 2024 Sep 12;4(9):e0003475. doi: 10.1371/journal.pgph.0003475 (PMC11392347; doi:10.1371/journal.pgph.0003475)
Supplement: S1 Table — (DOCX) [file pgph.0003475.s003.docx]

**Characteristics of the study population**

| **Variables** | **Total** | **Chitwan** | **Bhaktapur** | **Kathmandu** | **Lalitpur** | **Kaski** | **Morang** | **Sunsari** | **Rupendehi** |
| --- | --- | --- | --- | --- | --- | --- | --- | --- | --- |
|  |  |  |  |  |  |  |  |  |  |
| **Municipality** |  |  |  |  |  |  |  |  |  |
| Rural | 64 | - | - | - | 9 | 13 | 38 | - | 4 |
| Urban | 638 | 66 | 35 | 197 | 45 | 43 | 69 | 88 | 95 |
|  |  |  |  |  |  |  |  |  |  |
| **Gender** |  |  |  |  |  |  |  |  |  |
| Male | 332 | 28 | 9 | 81 | 21 | 18 | 63 | 43 | 69 |
| Female | 370 | 38 | 27 | 116 | 32 | 38 | 44 | 45 | 30 |
|  |  |  |  |  |  |  |  |  |  |
| **Marital status** |  |  |  |  |  |  |  |  |  |
| Unmarried | 218 | 24 | 17 | 69 | 22 | 12 | 30 | 26 | 18 |
| Married | 482 | 42 | 18 | 126 | 32 | 44 | 77 | 62 | 81 |
|  |  |  |  |  |  |  |  |  |  |
| **Main occupation** |  |  |  |  |  |  |  |  |  |
| Blue collar | 122 | 8 | 8 | 28 | 9 | 8 | 26 | 22 | 13 |
| White collar | 420 | 54 | 14 | 114 | 28 | 31 | 65 | 46 | 68 |
| Others | 125 | 4 | 8 | 39 | 17 | 11 | 13 | 18 | 15 |
|  |  |  |  |  |  |  |  |  |  |
| **Age (years)** |  |  |  |  |  |  |  |  |  |
| <20 | 33 | 3 | - | 4 | 1 | 1 | 7 | 9 | 8 |
| 20-30 | 260 | 38 | 18 | 77 | 23 | 16 | 43 | 25 | 20 |
| 31-40 | 159 | 17 | 4 | 39 | 15 | 13 | 25 | 18 | 28 |
| 41-50 | 138 | 6 | 4 | 50 | 10 | 11 | 20 | 17 | 20 |
| >50 | 109 | 2 | 9 | 26 | 5 | 15 | 12 | 18 | 22 |
|  |  |  |  |  |  |  |  |  |  |
| **Education of respondent** |  |  |  |  |  |  |  |  |  |
| No Education | 55 | 1 | 10 | 18 | 5 | 7 | 5 | 9 | - |
| Literate | 23 | 1 | - | 5 | 1 | 1 | 5 | 5 | 5 |
| Basic Education | 89 | 4 | 4 | 22 | 4 | 8 | 15 | 7 | 25 |
| Secondary Education | 231 | 19 | 1 | 49 | 19 | 16 | 52 | 37 | 38 |
| Undergraduate | 232 | 36 | 18 | 74 | 17 | 15 | 21 | 22 | 29 |
| Graduate and above | 69 | 5 | 2 | 29 | 7 | 8 | 9 | 8 | 1 |
|  |  |  |  |  |  |  |  |  |  |
| **Income-Nepalese rupees (NPR)** |  |  |  |  |  |  |  |  |  |
| <5000 | 29 | 1 | - | 2 | 1 | 5 | 6 | 7 | 7 |
| 5000-10,000 | 49 | - | 1 | 8 | 4 | 2 | 17 | 6 | 11 |
| 10,000-15,000 | 61 | - | 4 | 15 | 4 | 1 | 16 | 7 | 14 |
| 15,000-20,000 | 106 | 5 | 4 | 24 | 7 | 4 | 28 | 10 | 24 |
| >20,000 | 454 | 60 | 26 | 146 | 38 | 44 | 40 | 58 | 42 |
